# An integrated multi-omics analysis of the NK603 Roundup-tolerant GM maize reveals metabolism disturbances caused by the transformation process

**DOI:** 10.1038/srep37855

**Published:** 2016-12-19

**Authors:** Robin Mesnage, Sarah Z. Agapito-Tenfen, Vinicius Vilperte, George Renney, Malcolm Ward, Gilles-Eric Séralini, Rubens O. Nodari, Michael N. Antoniou

**Affiliations:** 1 Gene Expression and Therapy Group, King’s College London, Faculty of Life Sciences & Medicine, Department of Medical and Molecular Genetics, 8th Floor, Tower Wing, Guy’s Hospital, Great Maze Pond, London SE1 9RT, United Kingdom; 2 Genøk, Center for Biosafety, The Science Park, P.O. Box 6418 Tromsø 9294, Norway; 3 CropScience Department, Federal University of Santa Catarina, Rod. Admar Gonzaga 1346, 88034-000 Florianópolis, Brazil; 4 Proteomics Facility, King’s College London, Institute of Psychiatry, London SE5 8AF, United Kingdom; 5 University of Caen, Institute of Biology, EA 2608 and Network on Risks, Quality and Sustainable Environment, MRSH, Esplanade de la Paix, University of Caen, Caen 14032, Cedex, France

## Abstract

Glyphosate tolerant genetically modified (GM) maize NK603 was assessed as ‘substantially equivalent’ to its isogenic counterpart by a nutrient composition analysis in order to be granted market approval. We have applied contemporary in depth molecular profiling methods of NK603 maize kernels (sprayed or unsprayed with Roundup) and the isogenic corn to reassess its substantial equivalence status. Proteome profiles of the maize kernels revealed alterations in the levels of enzymes of glycolysis and TCA cycle pathways, which were reflective of an imbalance in energy metabolism. Changes in proteins and metabolites of glutathione metabolism were indicative of increased oxidative stress. The most pronounced metabolome differences between NK603 and its isogenic counterpart consisted of an increase in polyamines including N-acetyl-cadaverine (2.9-fold), N-acetylputrescine (1.8-fold), putrescine (2.7-fold) and cadaverine (28-fold), which depending on context can be either protective or a cause of toxicity. Our molecular profiling results show that NK603 and its isogenic control are not substantially equivalent.

The application of genetic engineering (GE) to modify edible crops is often advocated as one of the most important scientific advances to improve farming systems and feed the world in a more sustainable manner^1^. GE has been used to create crops adapted to abiotic stress, resistant to pathogens, with a longer shelf life, or with enhanced nutritional properties. However, commercialization of these traits is currently minor. Agricultural genetically modified (GM) crops are dominated by plants engineered to tolerate application of a herbicide or/and to produce their own insecticides^2^. A total of 180 million hectares of GM crops are currently cultivated worldwide on around 1.5 billion hectares constituting approximately 10% of global arable land^3^. Approximately 80% of GM crops have been modified to tolerate application of and thus accumulate glyphosate-based herbicide residues without dying in order to facilitate weed management.

Regulations for the release of genetically modified organisms (GMOs) of any kind in a country are covered by the national biosafety regulations of that nation. Guidance on risk assessment (RA) aim at identifying and avoiding adverse effects by early detection and proper evaluation of intended and potential unintended changes in a GMO. These should be detected and identified at early stages of RA, often referred to as “hazard identification”. Hazard identification is essential to the RA process as it sets the foundation of what is considered or observed in later steps in the risk assessment process^4^. In the US, the Food and Drug Administration considers GM technology as an extension of conventional breeding and GMO crops are deregulated once nutritional and compositional “substantial equivalence” is demonstrated^5^. The set of parameters and analyses necessary to declare a GMO as substantially equivalent to its conventional counterpart is still vague and focuses on a restricted set of compositional variables, such as the amounts of protein, carbohydrate, vitamins and minerals. GMOs are then declared substantially equivalent when sufficient similarities appear for those selected variables^6^. Remarkably, while a majority of GMO crops have been modified to withstand and thus accumulate a herbicide without dying, analysis for residues for such pesticides are neglected in compositional assessment^7^.

Recent technologies used to ascertain the molecular compositional profile of a system, such as transcriptomics, proteomics, metabolomics, epigenomics and mirnomics, collectively referred to as “*omics* technologies”, are used extensively in basic and applied science^8^. Comparative *omics* analyses have been performed comparing GMO crops and their isogenic counterpart. A number of them have shown metabolic disturbances from potential unintended effects of the GM transformation process in Bt maize^9^,^10^,^11^,^12^, glyphosate-tolerant soybean^13^,^14^,^15^, potato^16^, cotton^17^ and rice^18^. However, these studies do not report consistent or coherent results, which can be explained by the use of a variety of genetic backgrounds and/or different growth conditions, as well as variations in the technologies and threshold levels applied^19^. Indeed, the majority of authors of these types of studies conclude that the statistically significant changes observed between the conventional and the GM varieties are not biologically significant because they fall into the range of variations obtained in the comparisons between different conventionally-bred varieties, and under different environmental conditions^11^. However, other authors conclude that observed differences could reflect biologically significant, GM transformation process induced changes in protein profiles^12^ or metabolism^20^ when appropriate near-isogenic controls were applied and test crops grown at the same time and location to avoid differences brought about by variable environmental conditions^20^. Currently, no regulatory authority requires mandatory untargeted molecular profiling *omics* analysis to be performed but some acknowledge their potential relevance for food and feed derived from GM plants with specific metabolic pathways modified, or in situations where a suitable comparator is not available^4^,^21^.

Despite being declared to be ‘substantially equivalent’, off target effects have been observed in non-target species for Bt toxin-producing GMO crops^22^,^23^,^24^. Additionally, laboratory animal feeding trials performed with some GM plants in comparison to the non-GM counterpart have been proposed to provide evidence of ill-health effects. Several laboratory studies consisting of 90-day feeding trials in rodents have been conducted to evaluate the safety of GMO crop consumption^25^,^26^. These investigations have frequently resulted in statistically significant differences in parameters reflective of disturbances in various organ systems and in particular liver and kidney biochemistry, but with interpretation of their biological significance, especially with respect to health implications, being controversial^27^,^28^,^29^. Such differences in outcome in such laboratory animal feeding studies could have multiple sources including the presence of GMO-associated pesticide residues^30^,^31^.

In an effort to provide insight into the substantial equivalence classification of a Roundup tolerant NK603 GM maize, we have performed proteomics and metabolomics analyses of NK603 (sprayed or unsprayed with Roundup) and isogenic maize kernels (Fig. 1). We used a TMT10plex™ isobaric mass tag labelling method and quantified proteins by Liquid chromatography-tandem mass spectrometry (LC-MS/MS). The metabolome profile was determined by ultrahigh performance liquid chromatography-tandem mass spectroscopy (UPLC-MS/MS). Altogether, our integrative analysis shows that the GM transformation process used to generate NK603 maize caused deep alterations in the proteome and metabolome profiles of this crop and results in marked metabolic changes. We conclude that NK603 maize is not compositionally equivalent to its non-GM isogenic counterpart as previously claimed.

## Results

The objective of this investigation was to obtain a deeper understanding of the biology of the NK603 GM maize by molecular profiling (proteomics and metabolomics) in order to gain insight into its substantial equivalence classification. We began by undertaking an unsupervised exploratory analysis of variance structure. We integrated metabolome and proteome profiles of the NK603, cultivated either with or without Roundup, and its isogenic counterpart, into a two-step multiple co-inertia analysis (MCIA) process. First, a one-table ordination method transforms each multidimensional dataset (hyperspaces) separately into comparable lower dimensional spaces by finding axes maximizing the sum of the variances of the variables. The resulting variance structure can be described by a PCA (Additional file 3). The results show a clear separation of each feed type (NK603, NK603+Roundup and control) in both platforms. Control samples had the most distinct proteome and metabolome profiles as observed in PCA plots.

In a second step, the variance structure analyses from metabolome and proteome profiles were combined into a single analysis (Fig. 2). This aims to find new axes on which the two hyperspaces are projected by maximizing the square covariance. Figure 2A shows the projection of metabolome and proteome profiles onto the first two principal components of MCIA. Absolute eigenvalues of these components are given by a bar plot (Fig. 2B). The transgenic feed samples NK603 and NK603+Roundup are separated from the non-transgenic control (Isogenic) along the first component (horizontal axis). This clustering accounts for most of the variation (percentage of explained variance of 56.7%). The NK603 maize sprayed with Roundup separates from the unsprayed NK603 maize on the second component (vertical axis, percentage of explained variance of 16.6%). The lines connecting the different dots are proportional to the divergence between the different variables of the dataset. A relatively high correlation is depicted by the short edges. It shows similar trends in metabolome and proteome profiles, and also between the two cultivations, indicating that the most variant sources of biological information were similar. The projection of individual protein or metabolites on a 2-dimensional space (Fig. 2C) showed a mix pattern indicating that no particular subsets of variables are driving the clustering of groups. Finally, Fig. 2D shows the pseudo-eigenvalues space. The proteome samples (blue and green dots) are highly weighted on the horizontal axis indicating that this dataset is the highest contributor of the clustering of the transgenic feed samples from the control. By contrast, the differences between the NK603 maize sprayed with Roundup and the unsprayed NK603 maize are mostly due to the composition of the metabolome since the latest has a high weight on the vertical axis (red and black dots) of the pseudo-eigenvalues space. The fold changes observed in the comparisons of the NK603 maize sprayed with Roundup, the unsprayed NK603 maize and the isogenic control corn were highly correlated between the two cultivations performed during two different growing seasons (Additional file 4). Overall, the MCIA shows that the GM transformation process was the major contributor to variation in the protein and metabolite profiles rather than environmental factors such as the spraying of a pesticide or the growing season.

We next conducted a statistical evaluation of the biological differences resulting from the GM transformation process, as well as from the spraying of Roundup, by pairwise comparisons in order to identify proteins and metabolites associated with possible metabolic alterations. The list of proteins and metabolites having their levels significantly disturbed is given in Additional files 5 and 6, respectively. Figure 3 shows the statistical significance of differential protein/metabolite levels by volcano plots along with respective fold changes. While only one protein is newly produced as a result of the transgene insertion, a total of 117 proteins and 91 metabolites have been altered in maize by the genetic transformation process and insertion of the EPSPS-CP4 cassette (Isogenic vs NK603 panel, Fig. 3). One protein (B4G0K5) and 31 metabolites had their expression significantly altered by the spraying of the Roundup pesticide (NK603 vs NK603+Roundup (R) panel, Fig. 3).

The NK603 maize has been engineered to express a modified version of the *Agrobacterium tumefaciens* strain EPSPS-CP4^32^. Two peptides (lAGGEDVADLR and gLGNASGAAVATHLDHR) from EPSPS-CP4^33^ were detected and quantified by undertaking a specific targeted data analysis (Fig. 4). Their location on EPSPS-CP4 is shown by Fig. 4C. Reporter ion intensities for EPSPS-CP4 peptides in the NK603+Roundup and the NK603 were on average respectively 7 and 10 times higher than in the isogenic control. The observed signal for the non-transgenic corn probably represents non-specific background noise since it does not contain the EPSPS-CP4 gene. This would be caused by the co-isolation of other peptides in the corresponding MS/MS experiment, which gives rise to low intensity reporter ions in the control channels.

We analysed the biological information contained in proteome profiles from the NK603 and its isogenic counterpart to see if they bear a signature representative of metabolic disturbances caused by the insertion of the transgene cassette and/or the expression of bacterial EPSPS-CP4. Among different pathway enrichment analysis software tested, String was chosen due to its in-house predictions and homology transfers, as well as its connection to many fine external database resources, and thus its ability to identify a larger number of proteins. Nevertheless, our interpretation remained limited by the quality of protein annotation in such databases. A total of 42.7% (50/117) and 35% (55/156) of the proteins respectively disturbed in the comparison to the unsprayed or the sprayed NK603 maize were uncharacterized or not annotated in the databases (Additional file 5).

Pathway enrichment analysis of differentially expressed proteins in NK603 and NK603+Roundup feed samples was mainly assigned to carbohydrate and energy metabolism (Table 1). Most of the proteins, including enzymes, associated with these pathways were overexpressed in GM samples (Additional file 5). An increased expression of some proteins involved in glycolysis (FDR adjusted p-value = 4.2e-7), and in particular in the synthesis of pyruvate from D-glyceraldehyde 3-phosphate can be indicative of an increased demand for energy. Among them, pyruvate kinase (B4F9G8), enolase (ENO1), and three glyceraldehyde-3-phosphate dehydrogenases (GAPC1, GAPC2, GAPC3) had their levels increased in NK603 maize. Interestingly, gene ontology terms related to metabolic responses to stress were enriched (FDR adjusted p-value = 1.5e-6) and some heat shock proteins (e.g., HSP82) have been overexpressed.

The comparison between Roundup-sprayed NK603 and control samples revealed a similar pattern to that observed in unsprayed samples. However, glutathione metabolism (KEGG ID 480) showed a significant alteration in sprayed NK603. The proteins assigned to that pathway, glutathione S-transferase 1 and 6-phosphogluconate dehydrogenase (P12653 and B4FSV6 respectively) were more abundant in sprayed samples while another glutathione transferase isoform GST-5 (A0A0B4J3E6) was less abundant. Additionally, the 1-Cys peroxiredoxin PER and the peroxidase were overexpressed. Although only one protein was statistically significantly altered in a pairwise comparison between NK603+Roundup and NK603 as the effect of Roundup herbicide spray alone, the protein B4G0K5 that has an identified conserved domain of Ricin-type β-trefoil lectin. The Ricin-type β-trefoil is a carbohydrate-binding domain found in a variety of molecules serving diverse functions such as enzymatic activity, inhibitory toxicity and signal transduction^34^.

The composition of the metabolome is shown in Additional file 6. The most pronounced differences between the NK603 GM maize and its isogenic counterpart mostly consisted of an increase in the amounts of numerous polyamines. The levels of N-acetyl-cadaverine (2.9-fold), N-acetylputrescine (1.8-fold), putrescine (2.7-fold) and cadaverine (28-fold) were increased in NK603. The metabolome profile also highlighted an impairment of energy metabolism. While metabolites from the first part of the TCA cycle had their levels increased (α-ketoglutarate by 1.65-fold and citrate by 1.49-fold), metabolites from the second part of the TCA cycle had their levels decreased (malate by 0.59-fold, fumarate by 0.60-fold, succinate by 0.80-fold). Additionally, while proteins associated with glycolysis were overexpressed, carbohydrate metabolism is depleted in several metabolites (glucuronate by 0.63-fold, glucose 1-phosphate by 0.56-fold, maltohexaose by 0.28-fold, maltopentaose by 0.51-fold). Differences due to the pesticide spray were subtle: phenylpropanoid such as 4-hydroxycinnamate (0.63-fold), ferulate (0.59-fold) and sinapate (2.9-fold) were significantly changed. While alterations of the shikimate pathway were not detected, intermediates from aromatic amino acid metabolism (PEP derived) had their level increased (phenyllactate by 1.60-fold, phenylpyruvate by 2.71-fold, N-acetylphenylalanine by 2.24-fold and xanthurenate by 1.82-fold). These changes could be indicative of an increase in amino acid catabolism. However, of note is that PEP itself was not detected in the analysis.

Table 2 provides pathway enrichment analysis of metabolites that were found to be statistically significantly altered in the pairwise comparisons. For the metabolome pathways analysis, the profile of NK603 and NK603+R showed a distinct pattern compared to the profiles observed in the proteome analysis. From the 10 most altered pathways, these two samples shared only five altered pathways and these suggest an alteration due to the GM transformation process. These pathways revealed an alteration in aspartate, pyruvate and phenylalanine amino acid downstream processes. The NK603 metabolome profile seems to differ from sprayed samples by fatty acid related pathways and choline, nicotinate and nicotinamide metabolism while sprayed samples showed alterations in serine metabolism and other sugar related metabolism.

The STITCH tool was used to provide a visualisation of predicted interactions of chemicals and proteins that might have a link to the transgene-associated EPSPS-CP4 pathway. The interaction network reveals that some proteins or metabolites altered in the NK603 maize are interacting with EPSPS (Fig. 5). The network formed by these proteins/metabolites is centred on some TCA cycle intermediates, among them, the α-ketoglutarate. One should note that EPSPS is using an energy metabolism intermediate (phosphenolpyruvate) as substrate. Overall, our data shows that the expression of a heterologous EPSPS in the NK603 maize is causing a deep alteration in the proteome and metabolome profiles of feed samples and thus resulting in a metabolic imbalance.

## Discussion

In this report we present the first *multi-omics* analysis of GM NK603 maize compared to a near isogenic non-GM counterpart. Based on analysis conducted by the developer Monsanto Company, NK603 maize was scored as ‘substantially equivalent’ to its isogenic control, which was a major contributor to this product being granted market approval for animal and human consumption in the European Union, United States, Brazil and several other nations. Although NK603 had comparable nutritional and compositional profiles when originally accessed by the developer company upon registration of their product, our analysis at a detailed, in-depth molecular profiling level shows that NK603 grains, with or without Roundup spraying during cultivation, are not equivalent to isogenic non-transgenic control samples (Fig. 2).

The concept of substantial equivalence has long being used in safety testing of GMO crops, but the term and the concept has no clear definition^35^. In 1993 the Organization for Economic Co-operation and Development (OECD) stated that the “*concept of substantial equivalence embodies the idea that existing organisms used as food, or as a source of food, can be used as the basis for comparison when assessing the safety of human consumption of a food or food component that has been modified or is new*”^36^. The vagueness of this term generates conflict among stakeholders to determine which compositional differences are sufficient to declare a GMO as non-substantially equivalent. However, the Codex Alimentarius Commission^37^ makes it clear that a safety assessment of a new food based on the concept of substantial equivalence *“does not imply absolute safety of the new product; rather, it focuses on assessing the safety of any identified differences so that the safety of the new product can be considered relative to its conventional counterpart.”* Thus, the concept of substantial equivalence should not be used as a proof of safety. However, it could be used as a first tier in risk assessment to detect any unintended effects of the GM transformation process. Unintended effects can be understood as the effects that go beyond the primary expected effects of the genetic modification, and represent statistically significant differences in the GMO compared with an appropriate control^38^. Unintended effects during transgenesis include rearrangements, insertion, or deletions during the genetic transformation or during the tissue culture stages of GMO development^39^,^40^. A comprehensive characterization of the GM plant at the molecular level could facilitate identification of unintended effects in GMO crops and could be used as a complementary analytical tool to existing safety assessment procedures^41^,^42^,^43^,^44^.

In general, our study design further highlights the importance of restricting comparison to the GMO crop and non-GMO isogenic comparator and cultivation of the two at the same location and season when the objective is to evaluate the effect of the GM transformation process. This is obligatory in order to reduce effects on plant metabolism arising from differing environmental conditions, which can make it difficult to attribute differences that are observed to the procedure of transgenesis. However, even though our experimental design takes into account the effect of the growing season, further experiments made under different environmental conditions would be needed to determine the full range of effects of the GM transformation process on NK603 phenotype. Indeed, virtually all traits are influenced by genotype–environment interactions. Neither genetic differences nor environmental variations alone can account for the production of a particular phenotypic variation. For example, a study of the expression of the transgene encoding a Bt toxin in the MON810 GM maize under different environmental conditions, has shown that the phenotype resulting from the GM transformation process is influenced by stressful environmental conditions^45^.

The increasing literature reporting application of *omics* methods to assess proteome, metabolome and transcriptome profiles in GMO crops shows strong evidence of distinct grain proteomes in other GM maize events, such as MON810 Bt insecticide producing maize^11^,^12^,^46^. Although the majority of studies have focused on insect-resistant maize (e.g., MON810 event) and most likely because this was the first GM maize to enter the food and feed market, there has also been one previous metabolomics study investigating NK603. Metabolite profiling of NK603 maize kernels were analyzed and approximately 3% of the metabolites detected showed statistically significant differences compared to the respective isogenic lines^47^. Two metabolites (γ-tocopherol and myo-inositol) were less abundant in NK603^47^. Interestingly, γ-tocotrienol and myo-inositol levels were also found to be significantly reduced in our study, and thus attributable to the genetic transformation. This suggests that some metabolic alterations are consistently reported despite a strong background triggered by environmental influence. In a study of two common MON810/non-GM variety pairs subjected to two farming practices (conventional and low-nitrogen fertilization), it was found that up to 37.4% of the variation was dependent upon the variety, 31.9% were the result of the fertilization treatment, and 9.7% was attributable to the GM character^48^.

Alterations can also be found in other plant tissues. For example, analysis of leaves of Brazilian varieties of MON810 Bt maize revealed a total of 32 differentially expressed proteins between GM and non-GM samples that were identified and assigned to carbohydrate and energy metabolism, genetic information processing and stress response^9^.

Our study revealed significant metabolome profile differences between NK603 that was either sprayed or not with Roundup during cultivation (Fig. 2). This was surprising since the single application of this herbicide was prior to development of the maize cobs. In addition, we did not detect glyphosate or AMPA residues in the test maize kernel samples (Additional File 1). This indicates that metabolic differences provoked by an early application of Roundup persisted throughout the life of the maize even in the absence of herbicide residues. At present we can only speculate as to the mechanisms that may explain these effects but they may have their basis in epigenetic programming of gene expression patterns with consequent longer term effects. The spraying of Roundup could have acted as a signal causing an alteration in gene expression patterns in the growing maize. A recent study that demonstrated marked epigenetic (DNA methylation) changes in *A thaliana* in response to treatment with carbendazim supports this possibility^49^. In addition, it has been demonstrated that epigenetic (DNA methylation and post-translational histone modification) patterns acquired in one cultivation can be transgenerationally inherited in an *A thaliana* model system^50^. However, further research would be needed to determine if epigenetic alterations provoked by pesticide exposure can hamper plant phenotypes across generations.

The maize kernels analysed in this study were previously used to feed laboratory animals that formed part of a chronic (2 year) study looking at potential toxic effects arising from the consumption of this NK603 Roundup-tolerant GM maize. A dry feed was formulated to contain 11%, 22%, or 33% of NK603 maize, cultivated either with or without Roundup application, or 33% of the near isogenic variety. Sprague Dawley rats fed for two years on these diets presented blood/urine biochemical changes indicative of an increased incidence of liver and kidney structure and functional pathology in the NK603-containing diet groups compared to non-GM controls^51^. Standard biochemical compositional analysis revealed no particular differences between the different maize types tested^51^. Metabolic disturbances observed in our study may help to understand the negative health effects suggested after the chronic consumption of this GM maize. Alterations in concentrations of metabolites in grains might be directly related to pathogenic effects due to some active compounds that are known to be toxic^52^. For instance, a soybean glycoprotein allergen (Gly m Bd 28 K fragment) was also found overexpressed in a proteomic study of Roundup Ready GM soybean seeds (MSOY 7575 RR event)^13^. In our study, cadaverin levels were significantly increased (Log2FC 4.81 for NK603 and 5.31 for NK603+Roundup). Cadaverin plays important roles in lysine biosynthesis^53^ and also glutathione metabolism^54^. Other similar biogenic amines, such as N-acetyl-cadaverine, N-acetylputrescine and putrescine were also found to be present at higher levels in NK603 in our investigation. Different polyamines have been reported to have different effects, which depend on various factors such as age, tissue or disease status^55^. In certain contexts some of these polyamines have been found to be protective whereas in other situations they can be a cause of toxicity. On the one hand, toxicological effects such as nausea, headaches, rashes and changes in blood pressure are provoked by the consumption of foods with high concentrations of polyamines^56^. Putrescine and cadaverine have been reported as potentiators of the effects of histamine, and both have been implicated in the formation of carcinogenic nitrosamines with nitrite in meat products^57^. On the other hand, certain polyamines can also have beneficial anti-inflammatory effects and have been found to be beneficial during aging in some rodent model systems^58^. Noticeably, these polyamines were not measured in the first compositional analysis of NK603 maize performed for regulatory purposes^32^. Overall, whether the increased levels of cadaverine and putrescine found in the NK603 maize samples can account for the signs of potential negative health effects upon its consumption by rats, as implied by the blood/urine biochemical analysis^33^, needs to be further analyzed in experiments using more quantitative methods.

Our results suggest that expression of the EPSPS-CP4 transgene alters the oxidative environment in cells, and the increased levels of antioxidant enzymes are likely to be a response to oxidative burst by reactive oxygen species (ROS) in order to maintain proper physiological function. Glutathione metabolism was significantly altered in the NK603 when Roundup was sprayed during cultivation. Glutathione is known to be an important antioxidant in most living organisms, preventing damage to important cellular components caused by several environmental pollutants, including agrochemicals^59^. Plant glutathione S-transferases (GSTs) are also widely known for their role in herbicide detoxification^60^. Enzymes involved in combating reactive oxygen species, ascorbate peroxidase, glutathione reductase, and catalase are expressed at a higher level in transgenic soybean seeds^14^. Levels of ROS and other free radicals in GM food and feed would have to be monitored and quantified by further experiments in order to conclude on their potential impact on the agronomic performances of the plant. Additionally, it is known that polyamines are typically elevated in plants under abiotic stress conditions^61^. Typically, when cellular polyamine content increases, the levels of hydrogen peroxide also increases, activating antioxidant systems. Unintended effects of the inserted EPSPS-CP4 transgene was linked to energy metabolism disturbances in other studies^13^,^14^,^15^. It can be hypothesized that the plant is searching for a new equilibrium to maintain heterologous EPSPS-CP4 metabolism within levels that can be tolerated by the plant.

Glyphosate, the active ingredient of Roundup herbicide, inhibits the enzyme 5-enolpyruvylshikimate-3-phosphate synthase (EPSPS), which is the sixth enzyme of the shikimate pathway, and plays an essential role in the biosynthesis of aromatic amino acids and other aromatic compounds in plants^62^. The EPSPS has a binding site for phosphenolpyruvate (PEP) and it could be hypothesized that an overexpression of a heterologous EPSPS could provoke a metabolic imbalance by altering the metabolism of PEP. Alterations in intermediate metabolism are corroborated in our experiment by the fact that the network formed by altered proteins/metabolites is centred on some TCA cycle intermediates (Fig. 5) such as α-ketoglutarate. In fact, it is also known that EPSPS inhibition by glyphosate impairs carbon metabolism, in particular by inducing alternative respiration and aerobic fermentation^63^. In this latest study, the metabolic switch was explained by an accumulation of pyruvate. Thus, if EPSPS inhibition is able to alter intermediate metabolism, a comparable change in the opposite direction could be expected as a result of EPSPS overexpression.

This study is the first and most detailed *multi-omics* characterization of a widely commercialized GMO crop and its isogenic counterpart. In conclusion, our integrative statistical and bioinformatics analysis allowed us to suggest a mechanistic link between the proteome and metabolome alterations observed and the insertion of a particular transgene. The transformation process and the resulting expression of a transgenic protein cause a general disturbance in the GM plant and it is clear that NK603 maize is markedly different from its non-GM isogenic line at the proteome and metabolome levels. In addition, our data correlates with previous studies, which observed higher amounts of ROS that act as free-radicals promoting oxidative stress in those transgenic plant materials. We also confirm a metabolic imbalance in energy and carbohydrate metabolism. Although a clear mechanistic link between alterations in the GM feed and the possible health effects following long-term consumption of this product remains to be established, the evidence we present clearly shows that NK603 and non-GM isogenic maize are not substantially equivalent and the nutritional quality of GM feed might be hampered by metabolic imbalances related to plant energy and stress metabolism.

## Materials and Method

### Maize cultivation

The varieties of maize used in this study were DKC 2678 Roundup-tolerant NK603 (Monsanto Corp., USA), and its nearest isogenic non-transgenic control DKC 2675. These two types of maize were grown under similar normal conditions, in the same location and season, spaced at a sufficient distance to avoid cross-contamination. The site of cultivation consisted of an imperfectly drained field with a coarse loam surface texture and fine loam subsoil. A typical soil compositional analysis is provided in Additional File 1. The maize cultivation rows were spaced 75 cm apart, with approximately 30 cm between planted seeds (78,000 seeds/ha). One pass of the seeder included 4 rows of corn. To avoid edge effects in the field, 2 passes (8 rows) of DKC 2575 (isogenic) were planted as a buffer zone. DKC 2678 (NK603) and DKC 2575 (isogenic) were planted ~85 m apart. Half of the DKC 2678 received the treatment with Roundup WeatherMax.

Fertilization was performed with 26 T/ha liquid dairy manure, 100 kg/ha of 30-0-10 fertilizer was broadcast at planting, and 150 kg/ha of 18-46-0 fertilizer banded with the seed. The corn was harvested when the moisture content was less than 30%. All corn varieties were hand harvested by collecting ears in large tote bags to avoid cross contamination. The corn pickers were instructed to pick every ear of corn so as to avoid any risk of quality differentiation. Each corn variety was shelled (kernels removed from the cob) using a small threshing machine designed for this purpose. Each variety was dried in separate bulk drying bins to avoid any risk of cross contamination. The corn was dried at a low temperature (<30 °C) to avoid drying too rapidly and affecting feed quality. The corn was dried to <14% moisture before bagging.

**Figure 1:**
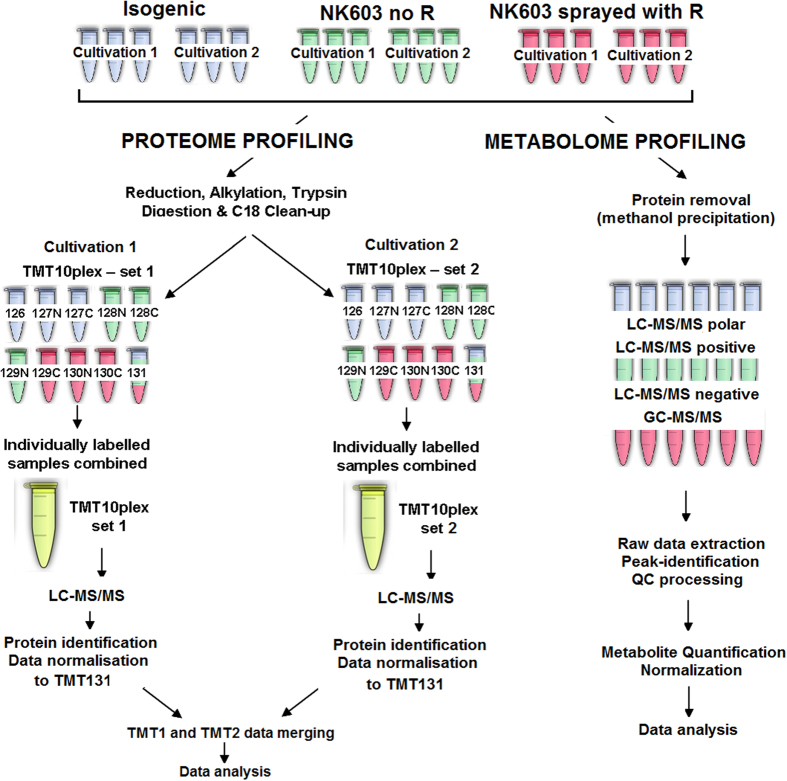
Flowchart of the experimental procedure. Harvested grains from NK603 GM maize cultivations, sprayed (NK603 + R) or not (NK603) with Roundup, were compared to their nearest isogenic non-transgenic control (Isogenic) grown under similar normal conditions. Two biological replicates were obtained by performing two cultivations at the same location in different years. Maize grains were analyzed by different mass spectrometry methods to determine proteome and metabolome profiles in 3 technical replicates.

**Figure 2:**
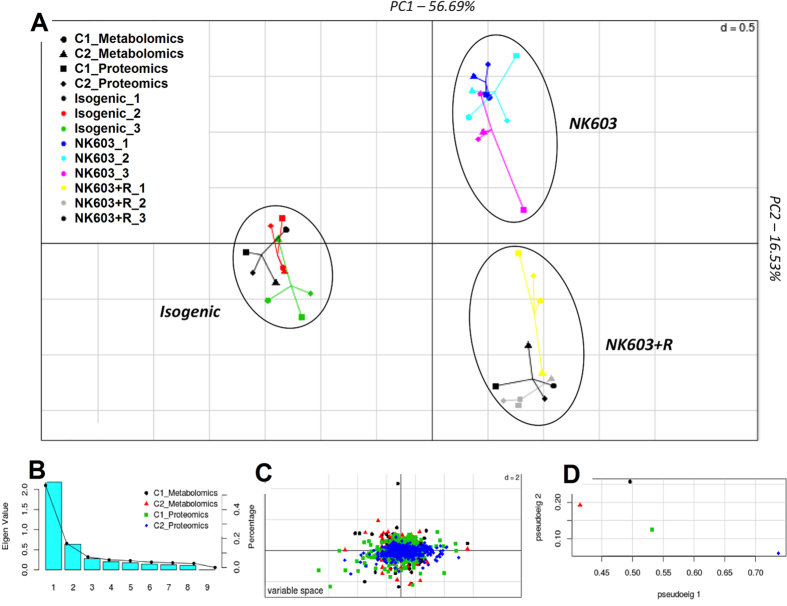
Integration of metabolome and proteome profiles of the NK603 maize and its near-isogenic counterpart into a multiple co-inertia analysis projection plot. (**A**) The first two axes of MCIA represent metabolome and proteomic datasets. Different shapes represent the different variables which are connected by lines, the length of these lines is proportional to the divergence between the data. Lines for each sample are joined at a common point at which the covariance derived from the MCIA analysis is maximal. (**B**) Pseudo-eigenvalue space showing the percentage of variance explained by each of the MCIA component. Each barplot represents the absolute eigenvalues. (**C**) Protein or metabolites (colored dots) are projected on a 2-dimensional space. In this panel, a protein or a metabolite that is particularly highly expressed in a maize variety will be located on the direction of this variety. (**D**) Pseudo-eigenvalues space of all datasets, indicating how much variance of an eigenvalue is contributed by the proteome or the metabolome for cultivations 1 and 2.

**Figure 3:**
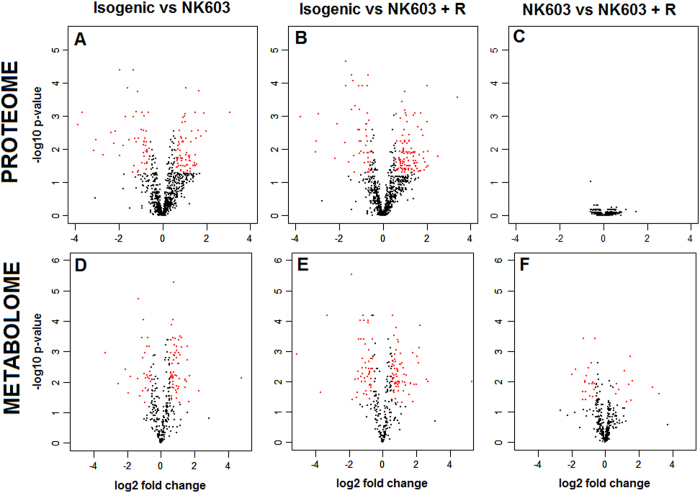
Volcano plots of the maize proteome and metabolome profiles. Volcano plots show the log 2 fold changes and the −log10 adjusted p-values in protein or metabolite level induced by the GM transformation process (isogenic vs NK603, isogenic vs NK603 + R) or by the pesticide spraying (NK603 vs NK603 + R). Data were selected at the cut off values adj-p < 0.05 and fold change >1.5. Red dots represent protein or metabolites having their level significantly altered in the different samples.

**Figure 4:**
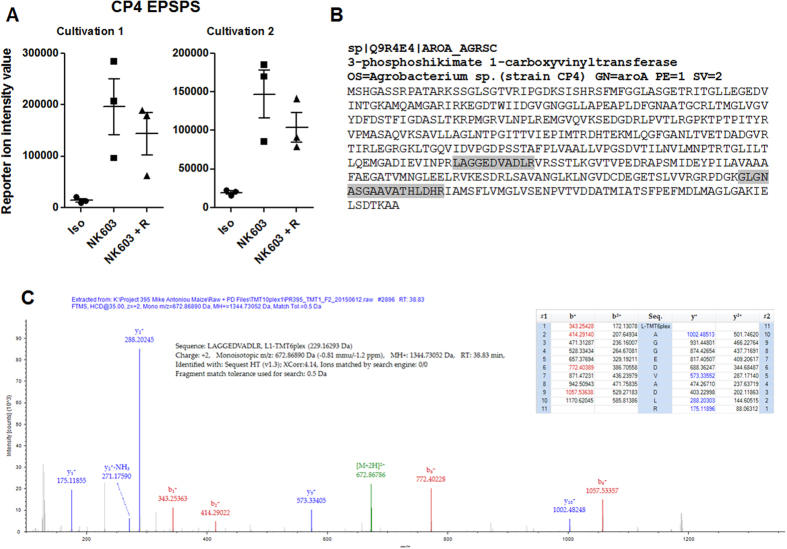
Mass spectrometric detection of CP4 EPSPS in the NK603 genetically modified maize. Two different peptides from Agrobacterium sp. 3-phosphoshikimate 1-carboxyvinyltransferase have been detected (gLGNASGAAVATHLDHR and lAGGEDVADLR) in all different samples allowing semi-quantitation (**A**) Reporter intensity ion values pertaining for CP4 EPSPS in the different samples of the two cultivations. (**B**) Localization of the peptides on the CP4 EPSPS (in grey) (**C**) Spectrum from the detection of the lAGGEDVADLR pertaining to Agrobacterium CP4 EPSPS (cultivation 1 of NK603).

**Figure 5:**
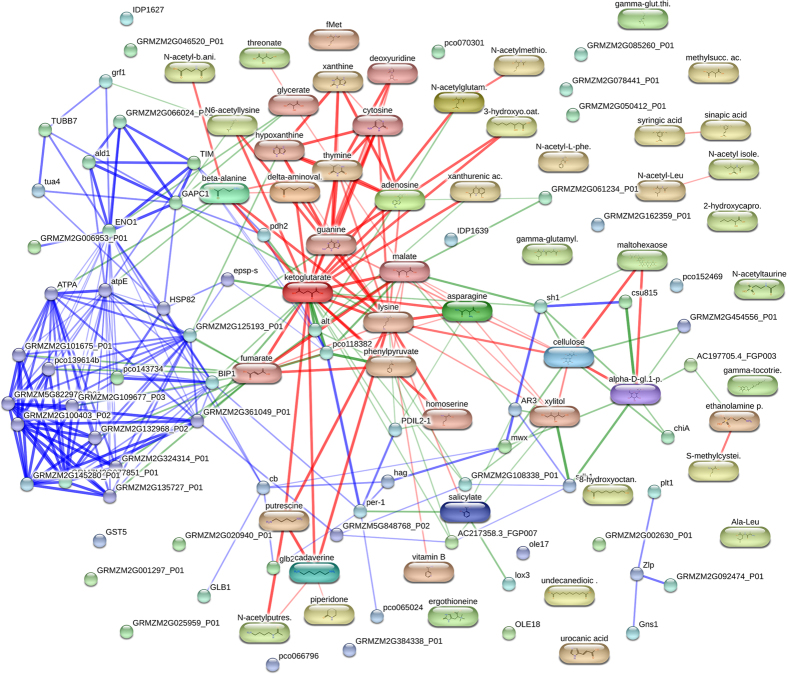
Interaction network of the metabolic effects resulting from the GM transformation process. The STITCH (‘Search Tool for Interacting Chemicals’) tool was used to provide a visualisation of the consequence of EPSPS insertion. The proteins and the metabolites which were found commonly deregulated in the two comparisons of NK603 and NK603 + R to its isogenic counterpart were used as input list. The top ten interaction partners with the highest scores as well as the maize EPSPS were included to reveal interactions.

The genetic nature, as well as the purity of the NK603 maize seeds and harvested material, was confirmed by quantitative PCR analysis of DNA samples. One field of NK603 was sprayed once with Roundup at 3 L ha^−1^ (WeatherMAX, 540 g/L of glyphosate, EPA Reg. 524–537) whilst another field of NK603 was not treated with Roundup. Test samples were produced by two cultivation cycles performed over two growing seasons. All maize samples were analysed for a total of 423 pesticide residues by SGS Institut Fresenius GmbH (Berlin, Germany), including glyphosate and its metabolite AMPA. No pesticide contaminants were detected in any of the samples (Additional file 2). All samples were maintained at −80 °C until processing for analysis. A schematic overview of our experimental design, sampling strategy and analytical approach is provided in Fig. 1.

### Proteome analysis

#### Sample preparation

Ground maize kernel samples were lysed in 8M lysis buffer (urea, NaCl, Tri-HCl, phosphatase and protease inhibitor) and their protein concentration calculated using a Nanodrop protein assay. Samples in triplicate were run through an SDS-PAGE 4–20% polyacrylamide gradient gel at 150 V. Excised gel bands were reduced with dithiothreitol (Sigma-Aldrich Ltd, Gillingham, Dorset, UK), alkylated with Iodoacetamide (Sigma-Aldrich Ltd) and digested with bovine sequencing grade trypsin (Roche, Penzberg, Germany; ref. 11418475001) at 37 °C for 18 hours. Subsequently extracted peptides were labelled with 60 mM TMT10plex Isobaric Label Reagents (ThermoFisher Scientific, Waltham, MA, USA; ref 90406) and the respective samples combined. Labelled peptides were then purified and extracted using Waters Sep-Pak Vac 3cc 200 mg tC18 cartridges, before being separated into 10 fractions by strong cation exchange (SCX) across an increasing salt concentration. The eluted peptide fractions were purified and extracted once again before being lyophilised for direct analysis by liquid chromatography-tandem mass spectrometry (LC-MS/MS).

#### Liquid chromatography-tandem mass spectrometry

Fractionated samples were resuspended in 100 μl of 50 mM ammonium bicarbonate and 10 μl of each of the 10 fractions was loaded onto a 50 cm EASY-spray column (ThermoFisher Scientific). Quantitative analysis was performed using the Orbitrap Velos-Pro mass spectrometer (ThermoFisher Scientific) in positive ion mode. The peptides were separated by gradient elution, from 5–80% 0.1% trifluoroacetic acid in acetonitrile (5–40% from 0–100 minutes, 40–80% from 100–110 minutes), at a flow rate of 300 nl/min. Mass spectra (m/z) ranging from 400–1600 Daltons was acquired at a resolution of 60,000 and the 10 most intense ions were subjected to MS/MS by HCD fragmentation with 35% collision energy.

#### Data processing

Protein identification was performed with Proteome Discoverer 1.4. Raw files were imported and searched against the UniProtKB/Swiss-Prot Database using Sequest for Proteome Discoverer. Raw files for all fractions were merged together in a single file search for each of the two TMT10plex sets. Precursor mass tolerance for the searches was set at 20ppm and fragment mass tolerance at 0.8ppm. The taxonomy selected was *Zea mays* and three enzymatic mis-cleavages were allowed. Dynamic modifications selected on the search were Oxidation/+15.995 Da (M) and Deamidated/+0.984 (N, Q) and static modifications were Carbamidomethyl/+57.021 Da (C), TMT10plex/229.163 Da (K), TMT10plex/229.163 Da (Any N-terminus). Only peptides with TMT reporter ion signal intensities for all ten samples were used for further bioinformatics analysis.

#### Metabolome analysis

The metabolome analysis was performed by Metabolon Inc. (Durham, NC, USA) as previously described^64^. Ground maize kernel samples were prepared using the automated MicroLab STAR^®^ system from Hamilton Company (Reno, NV, USA). Several recovery standards were added prior to the first step in the extraction process for QC purposes. In order to remove protein, dissociate small molecules bound to protein or trapped in the precipitated protein matrix, and to recover chemically diverse metabolites, proteins were precipitated with methanol under vigorous shaking for 2 min (Glen Mills GenoGrinder 2000) followed by centrifugation. The resulting extract was divided into five fractions: two for analysis by two separate reverse phase (RP)/UPLC-MS/MS methods with positive ion mode electrospray ionization (ESI), one for analysis by RP/UPLC-MS/MS with negative ion mode ESI, one for analysis by HILIC/UPLC-MS/MS with negative ion mode ESI, and one sample was reserved for backup. Samples were placed briefly on a TurboVap^®^ (SOTAX Corp, Westborough, MA, USA) to remove the organic solvent. The sample extracts were stored overnight under nitrogen before preparation for analysis.

#### Ultrahigh Performance Liquid Chromatography-Tandem Mass Spectroscopy (UPLC-MS/MS) for metabolome analysis

All methods utilized a Waters ACQUITY ultra-performance liquid chromatography (Waters Corp, Milford, MA, USA) and a ThermoFisher Scientific Q-Exactive high resolution/accurate mass spectrometer interfaced with a heated electrospray ionization (HESI-II) source and Orbitrap mass analyser operated at 35,000 mass resolution. The sample extract was dried then reconstituted in solvents compatible to each of the four methods. Each reconstitution solvent contained a series of standards at fixed concentrations to ensure injection and chromatographic consistency. One aliquot was analyzed using acidic positive ion conditions, chromatographically optimized for more hydrophilic compounds. In this method, the extract was gradient eluted from a C18 column (Waters UPLC BEH C18-2.1 × 100 mm, 1.7 μm) using water and methanol, containing 0.05% perfluoropentanoic acid (PFPA) and 0.1% formic acid (FA). Another aliquot was also analysed using acidic positive ion conditions, however it was chromatographically optimized for more hydrophobic compounds. In this method, the extract was gradient eluted from the same afore mentioned C18 column using methanol, acetonitrile, water, 0.05% PFPA and 0.01% FA and was operated at an overall higher organic content. Another aliquot was analysed using basic negative ion optimized conditions using a separate dedicated C18 column. The basic extracts were gradient eluted from the column using methanol and water, however with 6.5 mM ammonium bicarbonate at pH 8. The fourth aliquot was analysed via negative ionization following elution from a HILIC column (Waters UPLC BEH Amide 2.1 × 150 mm, 1.7 μm) using a gradient consisting of water and acetonitrile with 10 mM ammonium formate, pH 10.8. The MS analysis alternated between MS and data-dependent MSn scans using dynamic exclusion. The scan range varied slighted between methods but covered 70–1000 m/z.

#### Metabolome data processing

A quality control value assessment was undertaken to determine instrument variability by calculating the median relative standard deviation (RSD) for the internal standards that were pre-mixed into each sample prior to injection into the mass spectrometer. This yielded a value of 3% for instrument variability. Overall process variability as determined by calculating the median RSD for all endogenous metabolites (that is, non-instrument standards) present in 100% of the samples gave a value of 7%. Raw data was extracted, peak-identified and QC processed using Metabolon’s hardware and software as previously described^65^. Metabolites were identified by automated comparison and curated by visual inspection for quality control using software developed at Metabolon^66^. Peaks were quantified using area-under-the-curve.

#### Integrative bioinformatics analysis

For plotting of results, a Principal Component Analysis (PCA) was first performed. The language and statistical environment R^67^ together with the ade4 package^68^ method was employed in order to explore the relationship between GM and non-GM varieties. Second, we performed a Multiple Co-Inertia Analysis (MCIA), using the language and statistical environment R together with the omicade4 package^69^, in order to integrate multiple *omics* datasets where the same tissue have been assayed multiple times (in this case, proteomics and metabolomics).

Pairwise Welch’s t-tests were performed, for bothproteomics and metabolomics datasets, for Isogenic *vs* NK603, Isogenic *vs* NK603+Roundup and NK603 *vs* NK603+Roundup comparisons. The resulting p-values were adjusted by the Benjamini-Hochberg multi-test adjustment method for the high number of comparisons. Volcano plots were also constructed in order to visualize the differences in metabolite and protein expression for each of the comparisons. The aforementioned tests and plots were performed using in-house R scripts. Pathway enrichment analysis of the proteomics dataset was conducted using the web tool STRING v10.0^70^. For the metabolomics data, due to a lack of well-annotated metabolome databases for maize, the pathway enrichment analysis was conducted as follows. First, enrichment scores (ES) for each pathways were determined using the following formula: ES = (k/m)/(n/N) where (# of significant metabolites in pathway(k)/total # of detected metabolites in pathway(m))/(total # of significant metabolites(n)/total # of detected metabolites(N)). Then, the statistical significance was assessed using a Fisher one-sided exact test. The STITCH v5.0 beta web tool^71^ was used to investigate metabolite-protein interactions on maize endogenous pathways. The list of disturbed proteins and metabolites, including the protein EPSPS, was uploaded and the metabolic networks was studied using STITCH v5.0 initial parameters.

## Additional Information

**How to cite this article**: Mesnage, R. *et al*. An integrated multi-omics analysis of the NK603 Roundup-tolerant GM maize reveals metabolism disturbances caused by the transformation process. *Sci. Rep.* **6**, 37855; doi: 10.1038/srep37855 (2016).

**Publisher's note:** Springer Nature remains neutral with regard to jurisdictional claims in published maps and institutional affiliations.

## Supplementary Material

Supplementary Information

Supplementary Dataset 1

Supplementary Dataset 2

Supplementary Dataset 3

Supplementary Dataset 4

Supplementary Dataset 5

Supplementary Dataset 6

## Figures and Tables

**Table 1:** Pathway enrichment analysis in proteome profiles of the maize samples.

	Term description	n	p-adj
**Isogenic × NK603**			
KEGG ID			
1110	Biosynthesis of secondary metabolites	12	6.2E-11
1200	Carbon metabolism	8	1.6E-10
1120	Microbial metabolism in diverse environments	8	5.8E-10
1100	Metabolic pathways	13	1.3E-09
1230	Biosynthesis of amino acids	7	5.7E-09
710	Carbon fixation in photosynthetic organisms	5	3.6E-08
10	Glycolysis/Gluconeogenesis	5	4.2E-07
51	Fructose and mannose metabolism	3	9.4E-05
30	Pentose phosphate pathway	3	9.4E-05
520	Amino sugar and nucleotide sugar metabolism	3	6.6E-04
GO ID			
GO:0008150	Biological process	16	1.3E-18
GO:0008152	Metabolic process	13	5.0E-15
GO:0005975	Carbohydrate metabolic process	8	1.9E-14
GO:0016052	Carbohydrate catabolic process	6	4.0E-14
GO:0009987	Cellular process	12	6.1E-14
GO:0044699	Single-organism process	11	8.4E-14
GO:1901135	Carbohydrate derivative metabolic process	7	5.3E-13
GO:1901575	Organic substance catabolic process	6	5.9E-13
GO:0044238	Primary metabolic process	10	2.8E-12
GO:0071704	Organic substance metabolic process	10	5.9E-12
**Isogenic × NK603 + R**			
KEGG ID			
1110	Biosynthesis of secondary metabolites	14	2.3E-12
1200	Carbon metabolism	9	1.6E-11
710	Carbon fixation in photosynthetic organisms	7	1.6E-11
1120	Microbial metabolism in diverse environments	9	6.7E-11
1100	Metabolic pathways	15	1.3E-10
1230	Biosynthesis of amino acids	8	5.4E-10
10	Glycolysis/Gluconeogenesis	6	2.1E-08
51	Fructose and mannose metabolism	3	1.9E-04
30	Pentose phosphate pathway	3	1.9E-04
480	Glutathione metabolism	3	1.0E-03
GO ID			
GO:0008150	Biological process	21	2.3E-25
GO:0008152	Metabolic process	16	1.2E-18
GO:0009987	Cellular process	15	2.1E-17
GO:0016052	Carbohydrate catabolic process	7	1.3E-16
GO:0005975	Carbohydrate metabolic process	9	3.3E-16
GO:0044699	Single-organism process	13	4.3E-16
GO:0044723	Single-organism carbohydrate metabolic process	8	3.0E-15
GO:1901575	Organic substance catabolic process	7	3.0E-15
GO:0006757	ATP generation from ADP	6	3.0E-15
GO:0072524	Pyridine-containing compound metabolic process	6	3.0E-15

Among different analytical software, String was chosen as it recognized a maximum number of proteins. The maize genome was used as a background list to calculate the p-values of each term. The 10 most enriched GO biological process terms and KEGG pathways (ranked by p-values) are presented. N, number of protein disturbed in each pathway; p-adj, fdr adjusted p-value.

**Table 2:** Pathway enrichment analysis in metabolome profiles of the maize samples.

	n/N	ES	p-value
Iso vs NK603 pathways			
Amines and polyamines	3/4	2.6	0.0370
Nicotinate and nicotinamide metabolism	3/4	2.6	0.0370
Fatty acid, Dicarboxylate	4/8	1.7	0.0797
Aspartate family (OAA derived)	10/27	1.3	0.0908
TCA cycle	3/6	1.7	0.1300
Dipeptide	1/17	0.2	0.1359
Free fatty acid	7/19	1.3	0.1557
Branched Chain Amino Acids (pyruvate derived)	5/13	1.3	0.1780
Phenylpropanoids	3/7	1.5	0.1914
Photorespiration	2/4	1.7	0.2192
Iso vs NK603 + R pathways			
Amino sugar and nucleotide sugar	7/8	2.3	0.0007
Serine family (phosphoglycerate derived)	7/10	1.8	0.0062
Dipeptide	10/17	1.5	0.0096
TCA cycle	4/6	1.7	0.0532
Branched Chain Amino Acids (pyruvate derived)	7/13	1.4	0.0536
Aspartate family (OAA derived)	12/27	1.2	0.0730
Benzenoids	2/2	2.6	0.0769
Phenylpropanoids	4/7	1.5	0.0978
Glycolysis	3/5	1.6	0.1342
Inositol metabolism	2/3	1.7	0.1885
NK603 vs NK603 + R pathways			
Phenylpropanoids	3/7	4.3	0.0180
Amino sugar and nucleotide sugar	3/8	3.8	0.0271
Fatty acid conjugate	1/1	10.1	0.0899
Branched Chain Amino Acids (pyruvate derived)	3/13	2.3	0.1012
Choline metabolism	1/2	5.1	0.1719
gamma-glutamyl	2/10	2.0	0.2238
Serine family (phosphoglycerate derived)	2/10	2.0	0.2238
Fatty acid amide	1/3	3.4	0.2467
Nicotinate and nicotinamide metabolism	1/4	2.5	0.3150
Glycolysis	1/5	2.0	0.3773

The 10 most altered pathways (ranked by p-values) are presented. The number of metabolites disturbed in each pathway (n) is compared to the total number of metabolites measured for the given pathway (N). Enrichment scores (ES) for each pathway are calculated as follow: ES = (number of significant metabolites in pathway/total number of detected metabolites in pathway)/(total number of significant metabolites/total number of detected metabolites). The p-values were calculated according to a one sided Fisher exact test.

## References

[b1] Van Montagu M. It is a long way to GM agriculture. Annu Rev Plant Biol 62, 1–23 (2011). 21314429 10.1146/annurev-arplant-042110-103906

[b2] Parisi C., Tillie P. & Rodriguez-Cerezo E. The global pipeline of GM crops out to 2020. Nat Biotechnol 34, 31–36 (2016). 26744975 10.1038/nbt.3449

[b3] James C. Global Status of Commercialized Biotech/GM Crops: 2015. ISAAA Brief 51 (2015).

[b4] AHTEG. Guidance Document on Risk Assessment of Living Modified Organisms. United Nations Environment Programme Convention for Biodiversity. http://www.cbd.int/doc/meetings/bs/mop-05/official/mop-05-12-en.pdf (Date of access:24/10/2016) (2010).

[b5] Benbrook C. Enhancements Needed in GE Crop and Food Regulation in the U.S. Frontiers in public health 4, 59 (2016). 27066473 10.3389/fpubh.2016.00059 PMC4814896

[b6] Millstone E., Brunner E. & Mayer S. Beyond substantial equivalence. Nature 401, 525–526 (1999). 10524614 10.1038/44006

[b7] Cuhra M. Review of GMO safety assessment studies: glyphosate residues in Roundup Ready crops is an ignored issue. Environmental Sciences Europe 27, 1–14 (2015).

[b8] Heinemann J. A., Kurenbach B. & Quist D. Molecular profiling–a tool for addressing emerging gaps in the comparative risk assessment of GMOs. Environ Int 37, 1285–1293 (2011). 21624662 10.1016/j.envint.2011.05.006

[b9] Agapito-Tenfen S. Z., Guerra M. P., Wikmark O. G. & Nodari R. O. Comparative proteomic analysis of genetically modified maize grown under different agroecosystems conditions in Brazil. Proteome science 11, 46 (2013). 24304660 10.1186/1477-5956-11-46 PMC4176129

[b10] Agapito-Tenfen S. Z. . Effect of stacking insecticidal cry and herbicide tolerance epsps transgenes on transgenic maize proteome. BMC Plant Biology 14, 1–19 (2014). 25490888 10.1186/s12870-014-0346-8 PMC4273480

[b11] Barros E. . Comparison of two GM maize varieties with a near-isogenic non-GM variety using transcriptomics, proteomics and metabolomics. Plant biotechnology journal 8, 436–451 (2010). 20132517 10.1111/j.1467-7652.2009.00487.x

[b12] Zolla L., Rinalducci S., Antonioli P. & Righetti P. Proteomics as a complementary tool for identifying unintended side effects occurring in transgenic maize seeds as a result of genetic modifications. J Proteome Res 7, 1850–1861 (2008). 18393457 10.1021/pr0705082

[b13] Brandao A. R., Barbosa H. S. & Arruda M. A. Image analysis of two-dimensional gel electrophoresis for comparative proteomics of transgenic and non-transgenic soybean seeds. J Proteomics 73, 1433–1440 (2010). 20123049 10.1016/j.jprot.2010.01.009

[b14] Barbosa H. S., Arruda S. C., Azevedo R. A. & Arruda M. A. New insights on proteomics of transgenic soybean seeds: evaluation of differential expressions of enzymes and proteins. Anal Bioanal Chem 402, 299–314 (2012). 21947011 10.1007/s00216-011-5409-1

[b15] Arruda S. C., Barbosa H. S., Azevedo R. A. & Arruda M. A. Comparative studies focusing on transgenic through cp4EPSPS gene and non-transgenic soybean plants: an analysis of protein species and enzymes. J Proteomics 93, 107–116 (2013). 23796491 10.1016/j.jprot.2013.05.039

[b16] Lehesranta S. J. . Comparison of tuber proteomes of potato varieties, landraces, and genetically modified lines. Plant Physiol 138, 1690–1699 (2005). 15951487 10.1104/pp.105.060152 PMC1176438

[b17] Wang L. . Comparative proteomics of Bt-transgenic and non-transgenic cotton leaves. Proteome science 13, 15 (2015). 25949214 10.1186/s12953-015-0071-8 PMC4422549

[b18] Gong C. Y., Li Q., Yu H. T., Wang Z. & Wang T. Proteomics insight into the biological safety of transgenic modification of rice as compared with conventional genetic breeding and spontaneous genotypic variation. J Proteome Res 11, 3019–3029 (2012). 22509807 10.1021/pr300148w

[b19] Ricroch A. E., Bergé J. B. & Kuntz M. Evaluation of Genetically Engineered Crops Using Transcriptomic, Proteomic, and Metabolomic Profiling Techniques. Plant Physiol 155, 1752–1761 (2011). 21350035 10.1104/pp.111.173609 PMC3091128

[b20] Manetti C. . A metabonomic study of transgenic maize (Zea mays) seeds revealed variations in osmolytes and branched amino acids. J Exp Bot 57, 2613–2625 (2006). 16831843 10.1093/jxb/erl025

[b21] EFSA. Guidance on selection of comparators for the risk assessment of genetically modified plants and derived food and feed. EFSA J 2149, doi: 10.2903/j.efsa.2011.2150 (2011).

[b22] Losey J. E., Rayor L. S. & Carter M. E. Transgenic pollen harms monarch larvae. Nature 399, 214, doi: 10.1038/20338 (1999). 10353241

[b23] Hilbeck A. & Schmidt J. E. U. Another view on Bt proteins – how specific are they and what else might they do? Biopesti Int 2, 1–50 (2006).

[b24] Hilbeck A., Meier M. & Trtikova M., Underlying reasons of the controversy over adverse effects of Bt toxins on lady beetle and lacewing larvae. Environmental Sciences Europe 24, doi: 10.1186/2190-4715-24-9 (2012).

[b25] Hammond B., Dudek R., Lemen J. & Nemeth M. Results of a 13 week safety assurance study with rats fed grain from glyphosate tolerant corn. Food Chem Toxicol 42, 1003–1014 (2004). 15110110 10.1016/j.fct.2004.02.013

[b26] Hammond B. . Results of a 90-day safety assurance study with rats fed grain from corn rootworm-protected corn. Food Chem Toxicol 44, 147–160 (2006). 16084637 10.1016/j.fct.2005.06.008

[b27] Spiroux de Vendomois J. . Debate on GMOs health risks after statistical findings in regulatory tests. Int J Biol Sci 6, 590–598 (2010). 20941377 10.7150/ijbs.6.590 PMC2952409

[b28] Seralini G.-E. . Genetically modified crops safety assessments: present limits and possible improvements. Environ Sci Eur 23, 10 (2011).

[b29] Doull J. . Report of an Expert Panel on the reanalysis by of a 90-day study conducted by Monsanto in support of the safety of a genetically modified corn variety (MON 863). Food Chem Toxicol 45, 2073–2085 (2007). 17900781 10.1016/j.fct.2007.08.033

[b30] Mesnage R. & Séralini G.-É. The Need for a Closer Look at Pesticide Toxicity during GMO Assessment, in Practical Food Safety: Contemporary Issues and Future Directions (eds Bhat R. & Gómez-López V. M.) (John Wiley & Sons, Ltd, Chichester, UK, doi: 10.1002/9781118474563.ch10. 2014).

[b31] Mesnage R. . Transcriptome profile analysis reflects rat liver and kidney damage following chronic ultra-low dose Roundup exposure. Environ Health 14, 70 (2015). 26302742 10.1186/s12940-015-0056-1 PMC4549093

[b32] Monsanto. Safety Assessment of Roundup Ready Corn Event NK603 http://www.monsanto.com/products/documents/safety-summaries/corn_pss_nk603.pdf (Date of access: 24/10/2016) (2002).

[b33] Ocana M. F., Fraser P. D., Patel R. K., Halket J. M. & Bramley P. M. Mass spectrometric detection of CP4 EPSPS in genetically modified soya and maize. Rapid Commun Mass Spectrom 21, 319–328 (2007). 17200978 10.1002/rcm.2819

[b34] Marchler-Bauer A. . CDD: NCBI’s conserved domain database. Nucleic Acids Res 43, D222–226 (2015). 25414356 10.1093/nar/gku1221 PMC4383992

[b35] National Academies of Sciences, E., and Medicine. Genetically Engineered Crops: Experiences and Prospects (Washington, DC: The National Academies Press doi: 10.17226/23395 2016). 28230933

[b36] OECD. Safety Considerations for Biotechnology: Scale-up of Crop Plants. Paris: OECD. https://www.oecd.org/env/ehs/biotrack/1958527.pdf (Date of access: 24/10/2016) (1993).

[b37] Codex Alimentarius Commission Guideline for the Conduct of Food Safety Assessment of Foods Using Recombinant DNA Plants. Doc CAC/GL 45-2003. Rome: World Health Organization and Food and Agriculture Organization. http://www.fao.org/input/download/standards/10021/CXG_045e.pdf (Date of access: 24/10/2016) (2003).

[b38] AHTEG Final Report of the Ad Hoc Technical Expert Group on Risk Assessment and Risk Management under the Cartagena Protocol on Biosafety. UNEP/CBD/BS/AHTEG-RA&RM/4/6. http://www.cbd.int/doc/meetings/bs/bsrarm-04/official/bsrarm-04-06-en.pdf (Date of access: 24/10/2016) (2012).

[b39] Latham J. R., Wilson A. K. & Steinbrecher R. A. The mutational consequences of plant transformation. Journal of biomedicine & biotechnology 2006, 25376 (2006). 16883050 10.1155/JBB/2006/25376 PMC1559911

[b40] Fonseca C. . *In vitro* culture may be the major contributing factor for transgenic versus nontransgenic proteomic plant differences. Proteomics 15, 124–134 (2015). 25283639 10.1002/pmic.201400018

[b41] Garcia-Canas V., Simo C., Leon C., Ibanez E. & Cifuentes A. MS-based analytical methodologies to characterize genetically modified crops. Mass spectrometry reviews 30, 396–416 (2011). 21500243 10.1002/mas.20286

[b42] Simó C., Ibáez C., Valdés A., Cifuentes A. & García-Cañas V. Metabolomics of Genetically Modified Crops. International Journal of Molecular Sciences 15, 18941–18966 (2014). 25334064 10.3390/ijms151018941 PMC4227254

[b43] Heinemann J. A. & El-Kawy O. A. Observational science in the environmental risk assessment and management of GMOs. Environ Int 45, 68–71 (2012). 22575805 10.1016/j.envint.2012.03.011

[b44] D’Alessandro A. & Zolla L. We are what we eat: food safety and proteomics. J Proteome Res 11, 26–36 (2012). 21992580 10.1021/pr2008829

[b45] Trtikova M., Wikmark O. G., Zemp N., Widmer A. & Hilbeck A. Transgene Expression and Bt Protein Content in Transgenic Bt Maize (MON810) under Optimal and Stressful Environmental Conditions. PLoS ONE 10, e0123011 (2015). 25853814 10.1371/journal.pone.0123011 PMC4390241

[b46] Vidal N., Barbosa H., Jacob S. & Arruda M. Comparative study of transgenic and non-transgenic maize (Zea mays) flours commercialized in Brazil, focussing on proteomic analyses. Food chemistry 180, 288–294 (2015). 25766830 10.1016/j.foodchem.2015.02.051

[b47] Frank T., Rohlig R. M., Davies H. V., Barros E. & Engel K. H. Metabolite profiling of maize kernels–genetic modification versus environmental influence. J Agric Food Chem 60, 3005–3012 (2012). 22375597 10.1021/jf204167t

[b48] Coll A. . Natural variation explains most transcriptomic changes among maize plants of MON810 and comparable non-GM varieties subjected to two N-fertilization farming practices. Plant Mol Biol 73, 349–362 (2010). 20349115 10.1007/s11103-010-9624-5

[b49] Li Z., Wang Z. & Li S. Gene chip analysis of Arabidopsis thaliana genomic DNA methylation and gene expression in response to carbendazim. Biotechnology letters 37, 1297–1307 (2015). 25700823 10.1007/s10529-015-1789-1

[b50] Hauser M.-T., Aufsatz W., Jonak C. & Luschnig C. Transgenerational epigenetic inheritance in plants. Biochimica et Biophysica Acta (BBA) - Gene Regulatory Mechanisms 1809, 459–468 (2011). 21515434 10.1016/j.bbagrm.2011.03.007 PMC4359895

[b51] Seralini G.-E. . Republished study: long-term toxicity of a Roundup herbicide and a Roundup-tolerant genetically modified maize. Environmental Sciences Europe 26, 14 (2014). 27752412 10.1186/s12302-014-0014-5 PMC5044955

[b52] Esco Working Group M. EFSA Compendium of botanicals that have been reported to contain toxic, addictive, psychotropic or other substances of concern. EFSA Supporting Publications 6, doi: 10.2903/j.efsa.2012.2663 (2009).

[b53] Tomar P. C., Lakra N. & Mishra S. N. Cadaverine: a lysine catabolite involved in plant growth and development. Plant signaling & behavior 8, doi: 10 4161/psb 25850 (2013). 10.4161/psb.25850 PMC4091120 23887488

[b54] Simon-Sarkadi L., Ludidi N. & Kocsy G. Modification of cadaverine content by NO in salt-stressed maize. Plant signaling & behavior 9, e27598 (2014). 24398894 10.4161/psb.27598 PMC4091336

[b55] Minois N., Carmona-Gutierrez D. & Madeo F. Polyamines in aging and disease. Aging (Albany NY) 3, 716–732 (2011). 21869457 10.18632/aging.100361 PMC3184975

[b56] Álvarez González M. Á., Calles-Enríquez M., Fernández García M. & Ladero Losada V. M. Toxicological Effects of Dietary Biogenic Amines. Curr Nutr Food Sci 6, 145–156 (2010).

[b57] Nebelin E., Pillai S., Lund E. & Thomsen J. On the formation of N-nitrosopyrrolidine from potential precursors and nitrite. IARC scientific publications 183–193 (1980). 7228251

[b58] Soda K., Dobashi Y., Kano Y., Tsujinaka S. & Konishi F. Polyamine-rich food decreases age-associated pathology and mortality in aged mice. Exp Gerontol 44, 727–732 (2009). 19735716 10.1016/j.exger.2009.08.013

[b59] Couto N., Wood J. & Barber J. The role of glutathione reductase and related enzymes on cellular redox homoeostasis network. Free Radical Biology and Medicine 95, 27–42 (2016). 26923386 10.1016/j.freeradbiomed.2016.02.028

[b60] Milligan A. S., Daly A., Parry M. A. J., Lazzeri P. A. & Jepson I. The expression of a maize glutathione S-transferase gene in transgenic wheat confers herbicide tolerance, both in planta and *in vitro*. Molecular Breeding 7, 301–315 (2001).

[b61] Minocha R., Majumdar R. & Minocha S. C. Polyamines and abiotic stress in plants: A complex relationship. Frontiers in Plant Science 5 (2014). 10.3389/fpls.2014.00175 PMC4017135 24847338

[b62] Boocock M. R. & Coggins J. R. Kinetics of 5-enolpyruvylshikimate-3-phosphate synthase inhibition by glyphosate. FEBS Letters 154, 127–133 (1983). 11968207 10.1016/0014-5793(83)80888-6

[b63] Armendariz O., Gil-Monreal M., Zulet A., Zabalza A. & Royuela M. Both foliar and residual applications of herbicides that inhibit amino acid biosynthesis induce alternative respiration and aerobic fermentation in pea roots. Plant Biol (Stuttg) 18, 382–390 (2016). 26560850 10.1111/plb.12412

[b64] Evans A. M. . High Resolution Mass Spectrometry Improves Data Quantity and Quality as Compared to Unit Mass Resolution Mass Spectrometry in High-Throughput Profiling Metabolomics. Metabolomics 4, 132 (2014).

[b65] DeHaven C. D., E. A., Dai H. & Lawton K. A. Software Techniques for Enabling High-Throughput Analysis of Metabolomic Datasets. Metabolomics, Dr Ute Roessner (Ed.), ISBN: 978-953-51-0046-1, InTech, doi: 10.5772/31277 (2012).

[b66] Dehaven C. D., Evans A. M., Dai H. & Lawton K. A. Organization of GC/MS and LC/MS metabolomics data into chemical libraries. Journal of cheminformatics 2, 9 (2010). 20955607 10.1186/1758-2946-2-9 PMC2984397

[b67] Team R. C. R: A language and environment for statistical computing. R Foundation for Statistical Computing, Vienna, Austria, 2015. URL http://www.R-project.org/ (Date of access: 24/10/2016) (2015).

[b68] Dray S. & Dufour A.-B. The ade4 Package: Implementing the Duality Diagram for Ecologists. *2007* 22, 20 (2007).

[b69] Meng C., Kuster B., Culhane A. C. & Gholami A. M. A multivariate approach to the integration of multi-omics datasets. BMC bioinformatics 15, 1–13 (2014). 24884486 10.1186/1471-2105-15-162 PMC4053266

[b70] Szklarczyk D. . STRING v10: protein-protein interaction networks, integrated over the tree of life. Nucleic Acids Res 43, D447–452 (2015). 25352553 10.1093/nar/gku1003 PMC4383874

[b71] Szklarczyk D. . STITCH 5: augmenting protein-chemical interaction networks with tissue and affinity data. Nucleic Acids Res 44, D380–384 (2016). 26590256 10.1093/nar/gkv1277 PMC4702904

