# Author Correction: An integrated multi-omics analysis of the NK603 Roundup-tolerant GM maize reveals metabolism disturbances caused by the transformation process

**DOI:** 10.1038/s41598-019-39021-8

**Published:** 2019-03-15

**Authors:** Robin Mesnage, Sarah Z. Agapito-Tenfen, Vinicius Vilperte, George Renney, Malcolm Ward, Gilles-Eric Séralini, Rubens O. Nodari, Michael N. Antoniou

**Affiliations:** 1 Gene Expression and Therapy Group, King’s College London, Faculty of Life Sciences & Medicine, Department of Medical and Molecular Genetics, 8th Floor, Tower Wing, Guy’s Hospital, Great Maze Pond, London, SE1 9RT United Kingdom; 2 Genøk, Center for Biosafety, The Science Park, P.O. Box 6418, Tromsø, 9294 Norway; 3 CropScience Department, Federal University of Santa Catarina, Rod. Admar Gonzaga 1346, 88034-000 Florianópolis, Brazil; 4 Proteomics Facility, King’s College London, Institute of Psychiatry, London, SE5 8AF United Kingdom; 5 University of Caen, Institute of Biology, EA 2608 and Network on Risks, Quality and Sustainable Environment, MRSH, Esplanade de la Paix, University of Caen, Caen, 14032 Cedex France

Correction to: *Scientific Reports* 10.1038/srep37855, published online 19 December 2016

This Article contains errors.

In the Methods section under the heading ‘Proteome analysis’, subheading ‘Data Processing’:

“Precursor mass tolerance for the searches was set at 20ppm and fragment mass tolerance at 0.8ppm.”

should read:

“Precursor mass tolerance for the searches was set at 20ppm and fragment mass tolerance at 0.8 Daltons.”

The Data Availability section was omitted from the Article and is included below.

## Data Availability

The mass spectrometry proteomics data have been deposited to the ProteomeXchange Consortium via the MassIVE partner repository [https://massive.ucsd.edu/ProteoSAFe/static/massive.jsp] with the dataset identifier PXD011363.

