# Supplementary material for: An integrated multi-omics analysis of the NK603 Roundup-tolerant GM maize reveals metabolism disturbances caused by the transformation process
Source: Sci Rep. 2016 Dec 19;6:37855. doi: 10.1038/srep37855 (PMC5171704; doi:10.1038/srep37855)
Supplement: Supplementary Information [file srep37855-s1.doc]

**An integrated multi-omics analysis of the NK603 Roundup-tolerant GM maize reveals metabolism disturbances caused by the transformation process**

Robin Mesnage^1#^, Sarah Z Agapito-Tenfen^2#^, Vinicius Vilperte^3^, George Renney^4^, Malcolm Ward^4^, Gilles-Eric Séralini^5^, Rubens O Nodari^3^, and Michael N Antoniou^1*^

^1^ Gene Expression and Therapy Group, King's College London, Faculty of Life Sciences & Medicine, Department of Medical and Molecular Genetics, 8th Floor, Tower Wing, Guy's Hospital, Great Maze Pond, London SE1 9RT, United Kingdom.

^2^ Genøk, Center for Biosafety, The Science Park, P.O. Box 6418 Tromsø 9294, Norway.

^3^ CropScience Department, Federal University of Santa Catarina, Rod. Admar Gonzaga 1346, 88034-000 Florianópolis, Brazil.

^4^ Proteomics Facility, King's College London, Institute of Psychiatry, London SE5 8AF, United Kingdom.

^5^ University of Caen, Institute of Biology, EA 2608 and Network on Risks, Quality and Sustainable Environment, MRSH, Esplanade de la Paix, University of Caen, Caen 14032, Cedex, France

^*^ Correspondence: [michael.antoniou@kcl.ac.uk](mailto:michael.antoniou@kcl.ac.uk)

^#^ Joint first authors

**Additional files**

**Additional file 1.** Soil composition report.

**Additional file 2.** List of pesticides analyzed. All maize samples were analysed for a total of 423 pesticide residues by SGS Institut Fresenius GmbH (Berlin, Germany), including glyphosate and its metabolite AMPA.

**Additional file 3.** PCA analysis of the proteome (A) and metabolome (B) profiles show a distinct separation between the NK603 sprayed with Roundup, the NK603 unsprayed, and their non-transgenic control.

**Additional file 4.** Correlations between the fold changes observed in the comparisons of the NK603 maize sprayed with Roundup, the unsprayed NK603 maize and the isogenic corn during two different growing seasons.

**Additional file 5.** List of proteins having their level significantly altered by the GM transformation process. The isogenic maize proteome was compared to both NK603 and NK603 + R proteome.

**Additional file 6.** List of metabolites having their level altered for all pair-wise comparisons. The isogenic maize metabolome was compared to both NK603 and NK603 + R metabolome. Additionally, the list of metabolites significantly altered by the spraying of Roundup on the NK603 maize is also presented. The p-values were calculated according to a one sided Fisher exact test.
