# Supplementary material for: An integrated multi-omics analysis of the NK603 Roundup-tolerant GM maize reveals metabolism disturbances caused by the transformation process
Source: Sci Rep. 2016 Dec 19;6:37855. doi: 10.1038/srep37855 (PMC5171704; doi:10.1038/srep37855)
Supplement: Supplementary Dataset 1 [file srep37855-s2.doc]

**An integrated multi-omics analysis of the NK603 Roundup-tolerant GM maize reveals metabolism disturbances caused by the transformation process**

Robin Mesnage^1#^, Sarah Z Agapito-Tenfen^2#^, Vinicius Vilperte^3^, George Renney^4^, Malcolm Ward^4^, Gilles-Eric Séralini^5^, Rubens O Nodari^3^, and Michael N Antoniou^1*^

**Additional File 1 – Soil composition report**

| pH level | 6.1 |
| --- | --- |
| Organic Matter (%) | 2.6 |
| P2O5 – phosphorus (kg/ha) | 233 |
| K2O – potassium (kg/ha) | 208 |
| Ca – calcium (kg/ha) | 2772 |
| Mg (kg/ha) | 582 |
| Na (kg/ha) | 112 |
| Sulfur (kg/ha) | 69 |
| Fe – iron (ppm) | 358 |
| Mn – manganese (ppm) | 95 |
| Cu – copper (ppm) | 2.14 |
| Zn – zinc (ppm) | 4 |
| B – boron (ppm) | 0.3 |
| Cation Exchange Capacity (meq/100gm) | 12 |
| Base saturation - K% | 1.8 |
| Ca % | 57.9 |
| Mg % | 20.2 |
| Na % | 2 |
| H % | 18 |
