# Supplementary material for: An integrated multi-omics analysis of the NK603 Roundup-tolerant GM maize reveals metabolism disturbances caused by the transformation process
Source: Sci Rep. 2016 Dec 19;6:37855. doi: 10.1038/srep37855 (PMC5171704; doi:10.1038/srep37855)

**An integrated multi-omics analysis of the NK603 Roundup-tolerant GM maize reveals metabolism disturbances caused by the transformation process**

Robin Mesnage^1#^, Sarah Z Agapito-Tenfen^2#^, Vinicius Vilperte^3^, George Renney^4^, Malcolm Ward^4^, Gilles-Eric Séralini^5^, Rubens O Nodari^3^, and Michael N Antoniou^1*^

**Additional file 3.** PCA analysis of the proteome (A) and metabolome (B) profiles show a distinct separation between the NK603 sprayed with Roundup, the NK603 unsprayed, and their non-transgenic control.


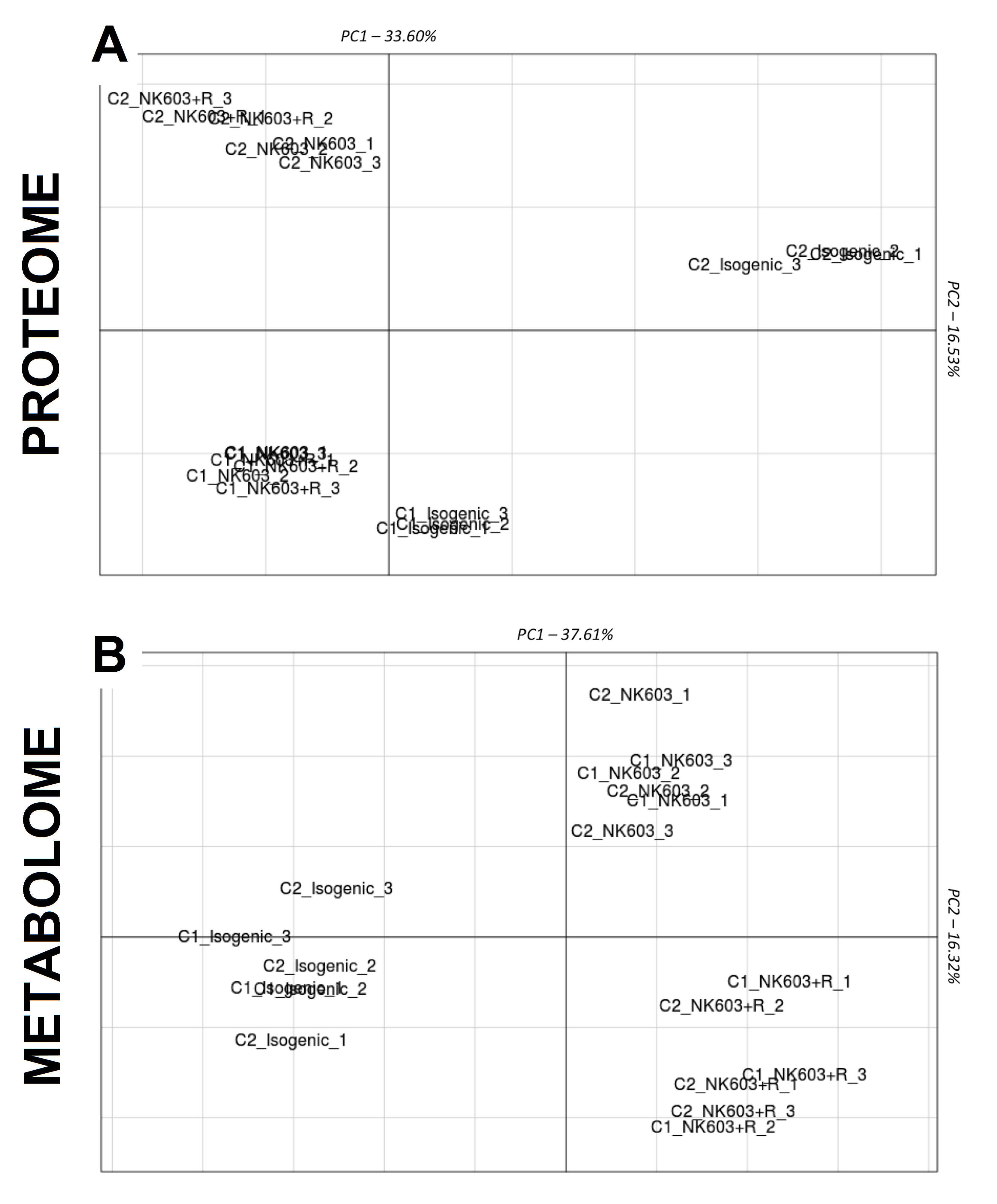

Supplement: Supplementary Dataset 3 [file srep37855-s4.doc]
