# Supplementary material for: An integrated multi-omics analysis of the NK603 Roundup-tolerant GM maize reveals metabolism disturbances caused by the transformation process
Source: Sci Rep. 2016 Dec 19;6:37855. doi: 10.1038/srep37855 (PMC5171704; doi:10.1038/srep37855)

**An integrated multi-omics analysis of the NK603 Roundup-tolerant GM maize reveals metabolism disturbances caused by the transformation process**

Robin Mesnage^1#^, Sarah Z Agapito-Tenfen^2#^, Vinicius Vilperte^3^, George Renney^4^, Malcolm Ward^4^, Gilles-Eric Séralini^5^, Rubens O Nodari^3^, and Michael N Antoniou^1*^

**Additional file 4.** Correlations between the fold changes observed in the comparisons of the NK603 maize sprayed with Roundup, the unsprayed NK603 maize and the isogenic corn during two different growing seasons


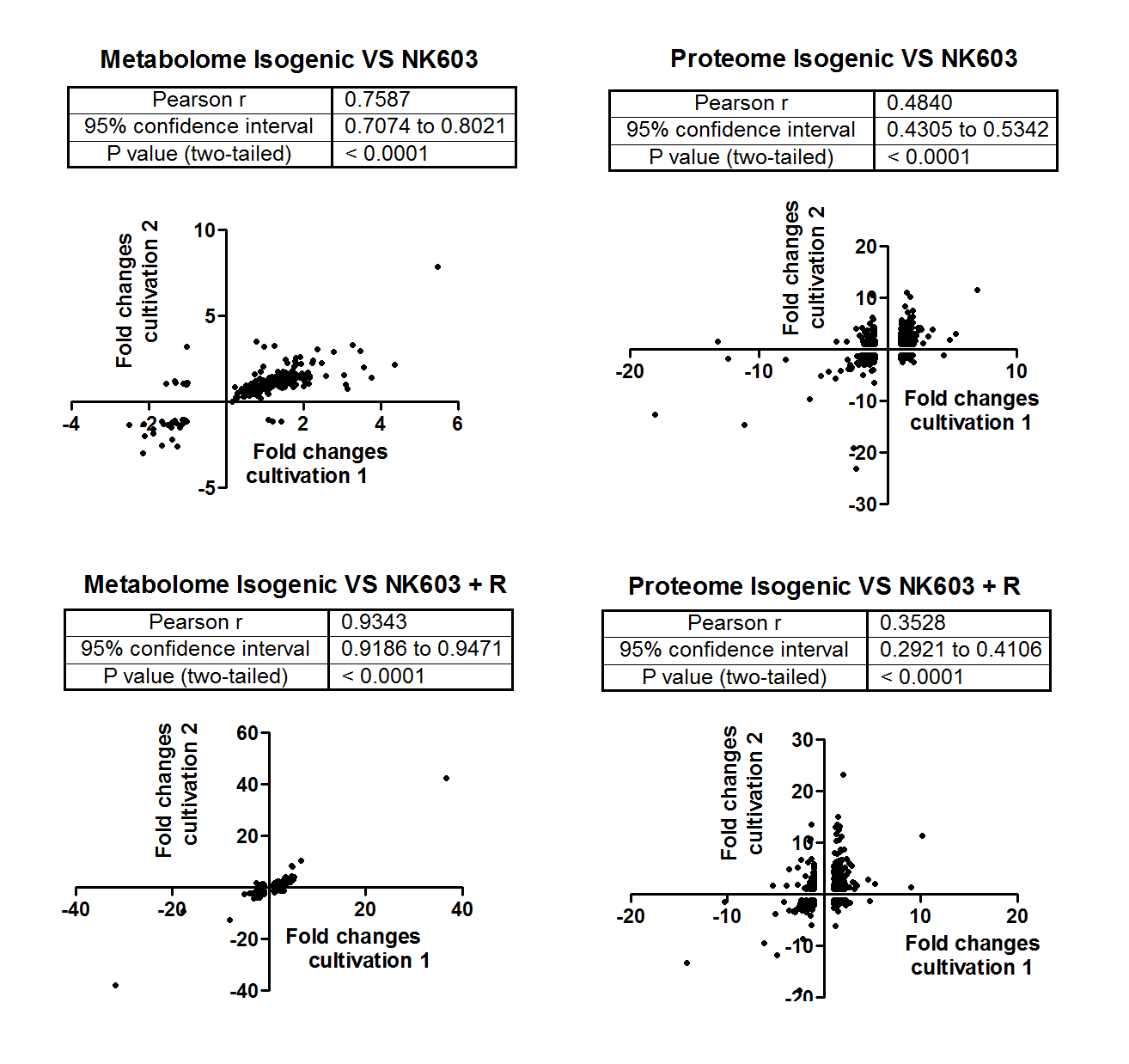

Supplement: Supplementary Dataset 4 [file srep37855-s5.doc]
